# Role of Dynamic Susceptibility Contrast Perfusion MRI in Glioma Progression Evaluation

**DOI:** 10.1155/2021/1696387

**Published:** 2021-02-09

**Authors:** Guanmin Quan, Kexin Zhang, Yawu Liu, Jia-Liang Ren, Deyou Huang, Weiwei Wang, Tao Yuan

**Affiliations:** ^1^Department of Medical Imaging, The Second Hospital of Hebei Medical University, Shijiazhuang, China; ^2^Department of Clinical Radiology, Kuopio University Hospital, Kuopio, Finland; ^3^GE Healthcare China, Beijing, China; ^4^Department of Radiology, Affiliated Hospital of Youjiang Medical University for Nationalities, Baise, China; ^5^Department of Radiology, Handan Central Hospital, Handan, China

## Abstract

Accurately and quickly differentiating true progression from pseudoprogression in glioma patients is still a challenge. This study aims to explore if dynamic susceptibility contrast- (DSC-) MRI can improve the evaluation of glioma progression. We enrolled 65 glioma patients with suspected gadolinium-enhancing lesion. Longitudinal MRI follow-up (mean 590 days, range: 210–2670 days) or re-operation (*n* = 3) was used to confirm true progression (*n* = 51) and pseudoprogression (*n* = 14). We assessed the diagnostic performance of each MRI variable and the different combinations. Our results showed that the relative cerebral blood volume (rCBV) in the true progression group (1.094, 95%CI: 1.135–1.636) was significantly higher than that of the pseudoprogression group (0.541 ± 0.154) (*p* < 0.001). Among the 18 patients who had serial DSC-MRI, the rCBV of the progression group (0.480, 95%CI: 0.173–0.810) differed significantly from pseudoprogression (-0.083, 95%CI: −1.138–0.620) group (*p*=0.015). With an rCBV threshold of 0.743, the sensitivity and specificity for discriminating true progression from pseudoprogression were 76.5% and 92.9%, respectively. The Cho/Cr and Cho/NAA ratios of the true progression group (2.520, 95%CI: 2.331–2.773; 2.414 ± 0.665, respectively) were higher than those of the pseudoprogression group (1.719 ± 0.664; 1.499 ± 0.500, respectively) ((*p*=0.001), (*p* < 0.001), respectively). The areas under ROC curve (AUCs) of enhancement pattern, MRS, and DSC-MRI for the differentiation were 0.782, 0.881, and 0.912, respectively. Interestingly, when combined enhancement pattern, MRS, and DSC-MRI variables, the AUC was 0.965 and achieved sensitivity 90.2% and specificity 100.0%. Our results suggest that DSC-MRI can significantly improve the diagnostic performance for identifying glioma progression. DSC-MRI combined with conventional MRI may promptly distinguish true gliomas progression from pseudoprogression when the suspected gadolinium-enhancing lesion was found, without the need for a long-term follow-up.

## 1. Introduction

Gliomas are the most common primary brain tumors in adults [1]. At present, the standard treatment for gliomas involves maximal safe tumor resection, followed by radiotherapy and concomitant and adjuvant chemotherapy with temozolomide. Recurrence and tissue response to the treatment or so-called pseudoprogression are both associated with a high incidence of new enhancement or enlargement of preexisting enhancement on contrast-enhanced T1-weighted (CE-T1WI) MR series, especially in patients with high-grade gliomas. Pseudoprogression lesions can appear both in the first three months and long after completion of radiation with an incidence of 9%–36% [2, 3]. The pseudoprogression lesions are caused by normal tissue response to treatment, whereas true progression is the result of tumor recurrence and needs salvage therapy [4]. Hence, it is essential to discriminate true progression from pseudoprogression as early as possible.

As a golden standard, histopathology is limited in clinical practice for the disadvantage of invasive sampling, sampling error, and high cost [5]. MRI is the most common modality of follow-up tool in patients with posttreatment glioma. However, the standard imaging protocol in the Response Assessment in Neuro-Oncology (RANO) criteria, including fluid-attenuated inversion recovery (FLAIR) and CE-T1WI, does not specifically require to discriminate the true progression from the pseudoprogression lesions [4, 6]. What is more, long-term follow-up should be made to discriminate true progression from pseudoprogression when new gadolinium-enhancing lesion or increase of the preexisting enhancing lesion is detected. Consequently, longitudinal follow-up MRI and various advanced functional MR techniques have been applied to obtain a definitive diagnosis. The long waiting time for a definitive diagnosis by means of follow-up MRI is a psychological torture to patients and may delay salvage therapy. On the other hand, in spite of promising results, the application of advanced functional MR techniques is limited for their vague results as well as not being a routine exam in the clinic [6–8].

Previous studies have shown that MR perfusion-weighted imaging, especially dynamic susceptibility contrast perfusion MRI (DSC-MRI), can reflect tumor angiogenesis which is valuable for glioma grading, estimation of prognosis, and differentiating tumor recurrence from treatment-related changes [5, 9, 10]. But there are only a few reports that assessed the impact of combined DSC-MRI and other MRI features on identifying the true tumor progression [5, 9, 11]. Since functional MR imaging techniques can reflect different pathological changes, such as diffusion-weighted imaging (DWI) for water molecule movement, and magnetic resonance spectroscopy (MRS) for neoplastic metabolites, we hypothesized that combination of DSC-MRI, DWI, and MRS can improve the accuracy for identification of true progression lesions among the gadolinium-enhancing lesions. Therefore, the objective of this study was to explore whether DSC-MRI can improve diagnostic performance of multiparametric MRI in the evaluation of progression in gliomas patients after standard treatment.

## 2. Materials and Methods

### 2.1. Patients

This study was approved by the Institutional Review Board. The informed consent was waived for its retrospective nature. A total of consecutive 65 glioma patients with suspicious gadolinium-enhancing lesion were recruited (36 males, 29 females; mean age, 46.5 ± 14.3 years), according to the following eligibility criteria: (1) gliomas were confirmed by histology; (2) gross-total resection of tumors [12]; (3) completed the standard treatment according to National Comprehensive Cancer Network (NCCN) guideline, including gross-total resection, radiation therapy and chemotherapy (CCRT) after surgery, and six cycles of adjuvant temozolomide [13]; (4) had standard clinical MRI before and after chemoradiotherapy; (5) gadolinium-enhancing lesion enlarged or presence of new gadolinium-enhancing lesion within the first month after chemoradiotherapy [6]; (6) had follow-up MRI at least 12 months after chemoradiotherapy; (7) had multi-parametric MR imaging when suspected gadolinium-enhancing lesion was found in the first follow-up MRI.

### 2.2. Conventional and Perfusion MRI

All studies were performed on a 3T MRI scanner (Achieva; Philips Medical System, Best, The Netherlands), using an 8-channel phased-array coil for acquisition. The conventional MR protocol included precontrast and postcontrast T1WI, T2WI, and FLAIR. The multi-parametric MR imaging protocol included T_1_WI, T_2_WI, FLAIR, DWI, MRS, and DSC-MRI. The parameters of DSC-MRI: perfusion-weighted gradient-echo echo-planar sequence; repetition time, 2000 ms; echo time, 40 ms; slice thickness, 6.5 mm; the data was acquired every one second for a total of 1 min 30 sec, with Gd-DTPA (Gadovist, Bayer Schering Pharma, Berlin, Germany, 0.2 ml/kg of body weight and maximum dose of 20 ml) injected with a MRI-compatible power injector at a rate of 3 ml/sec, followed by a 20-ml 0.9% saline flush using same flow rate.

### 2.3. Imaging Analysis

**Figure 1:**
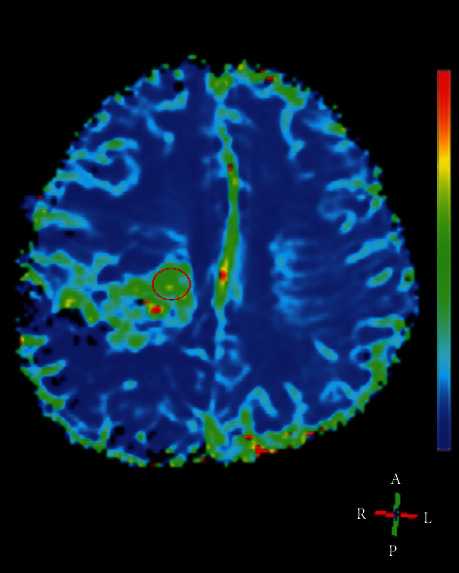
Example of region of interest (ROI) for measurement of CBV (the circle in red). This 37-year-old female patient with pathologically proven glioblastoma was ascribed to true progression group.

The imaging analysis and postprocessing were performed on a workstation (PHILIPS Extended MR WorkSpace 2.6.3.4). The enhancement patterns of residual cavity wall were divided into thin-linear (partial or entire wall enhancement with thickness <3 mm), thick-linear (partial or entire wall enhancement of 3–5 mm in thickness), and nodular wall enhancement (with nodular enhancement of 5–10 mm in thickness) patterns [14]. Cerebral blood volume (CBV) was calculated based on signal intensity-time curves in transverse *T*_2_^*∗*^-weighted sequence. We placed 3 circular ROIs in the region with the largest enhancement (Figure 1), and the mean CBV was calculated and used in quantitative analysis. We calculated the relative CBV (rCBV) of suspected gadolinium-enhancing lesion as well as ipsilateral normal tissues by dividing the CBV values of these regions with the CBV values of contralateral normal white matter [15]. The rCBV difference between two sequential perfusion imaging was calculated for those patients with longitudinal DSC-MRI examinations [10]. The apparent diffusion coefficient (ADC) was measured in the same regions as CBV measurements. The conventional and functional MRI characteristics were analyzed independently by two neuroradiologists (with 10 and 16 years of neuroradiology experience, respectively). When a disagreement existed, a consent was reached after consulting another neuroradiologist (with 25-year experience in neuroradiology). The outcome of the tumor was assessed according to the updated RANO criteria for gliomas [6, 16]. True progression was defined when patients with newly enhancement lesions or with increase the size of enhancement lesions continuously in the follow-up period after accomplishment of chemoradiotherapy. If there were stable or regressing enhancement lesions, the patients were defined as pseudoprogression [6].

### 2.4. Statistical Analysis

Statistical analysis utilized the software SPSS for windows release 25.0 (SPSS Inc., Chicago, IL, USA). Categorical variables were analyzed with log-rank test. The quantitative data between true progression and pseudoprogression groups, including rCBV, Cho/Cr, Cho/NAA, and ADC, were compared by using two-tailed Student's *t*-test when they were in non-normal distribution, or using Mann–Whitney U test when they were in non-normal distribution. The interobserver consistency between the two neuroradiologists was evaluated with intraclass correlation coefficient (ICC). The survival times were estimated with the Kaplan–Meier methods. Receiver operating characteristic (ROC) curve analysis was employed in determining the best cutoff values of rCBV and metabolite ratios in differentiating true progression and pseudoprogression by maximizing the sum of sensitivity and specificity. The diagnostic performance of all variables was measured as area under ROC curve (AUC). The level of significance was set at *p* < 0.05.

## 3. Results

**Figure 2:**
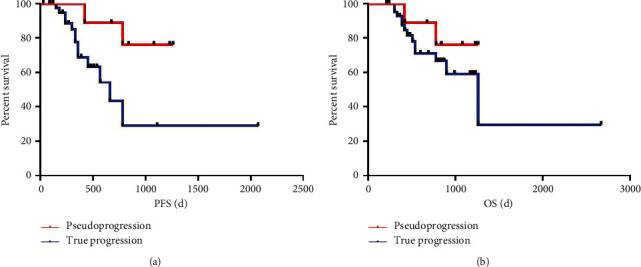
Kaplan–Meier survival curve for PFS and OS according to true progression and pseudoprogression. PFS, progression-free survival; OS, overall survival.

The median follow-up span was 590 days (range: 210–2670 days). The median progression-free survival (PFS) was 360 days [95% confidence interval (CI): 399–580 days] and median overall survival (OS) was 590 days (95% CI: 603–790 days) (Figure 2). 22 patients (33.846%) were dead during the follow-up period. The demographic and MR imaging characteristics of the 65 patients are summarized in Table 1. Fifty-one patients (28 males, 47.290 ± 14.380 years) were diagnosed as true progression (histologically confirmed in 3 cases) and fourteen patients (8 males, 43.640 ± 14.370 years) were diagnosed as pseudoprogression. There was no significant difference in patient age (*p*=0.403), radiation dose (*p*=0.615), histopathologic grade (*p*=0.451), and Karnofsky performance status (KPS) scores (*p*=0.154).

Among the patients with DWI data (54/65), there was no significant difference in ADC between two groups (*p*=0.067) in spite of lower ADC value for true progression group (Table 1). The Cho/Cr and Cho/NAA of true progression group (2.520, 95%CI: 2.331–2.773; 2.414 ± 0.665, respectively) were higher (*p* ≤ 0.002) than those of pseudoprogression group (1.719 ± 0.664; 1.499 ± 0.500, respectively) (Table 2), whereas there was no significant difference in NAA/Cr ratio between true progression (1.030 ± 1.100) and pseudoprogression groups (1.300 ± 0.750) (*p*=0.485). Using a cutoff Cho/Cr value of 2.475, a sensitivity 51.0% and specificity 92.9% were achieved; using a cutoff Cho/NAA value of 2.155, a sensitivity 64.7% and specificity 100.0% were achieved for separating the two groups.

The rCBV of normal brain tissue was 0.993 ± 0.106 (0.854–1.209). There was significant difference between the rCBV of normal brain tissue and that of true progression lesions (*p* < 0.001), so did between the normal brain tissue and pseudoprogression lesions (*p* < 0.001). The rCBV of true progression group (1.094, 95%CI: 1.135–1.636) was significantly higher than that of pseudoprogression group (0.541 ± 0.154) (*p* < 0.001) (Table 2). Among 65 patients, 18 patients (34.0%, 14 patients with true progression and 4 patients with pseudoprogression lesions) had serial DSC-MRI. Changes in rCBV at subsequent follow-up differed significantly (*p*=0.015) between true progression (0.480, 95%CI: 0.173–0.810) and pseudoprogression (−0.083, 95%CI: −1.138–0.620) groups. The between-group comparison revealed a significant difference between the pseudoprogression and the true progression groups corresponding to a large effect size (Cohen's *d* = 1.392). The ROC analysis showed that, using a cutoff rCBV value of 0.045, it achieved AUC 0.904, sensitivity 100.0%, and specificity 75.0% to separate the two groups.

**Figure 3:**
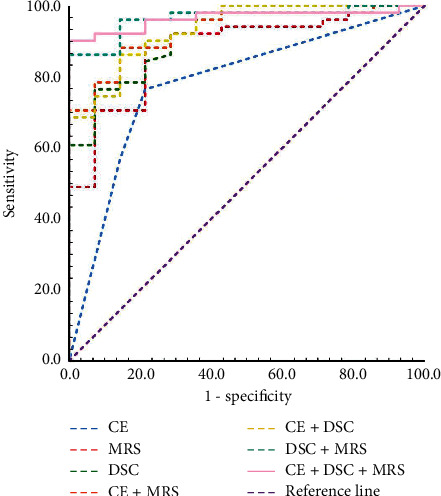
Comparison of ROC curve analyses for different MR variables and their combinations. The combination of CE + DSC + MRS presents the high diagnostic performance, and the AUC was 0.965 (95%CI 0.920–1.000), with significant level of *p* < 0.05 to any other variables and other combinations. CE, contrast enhancement pattern of residual cavity wall; MRS, Cho/Cr and Cho/NAA ratios; DSC, dynamic susceptibility contrast perfusion.

Figure 3 shows the ROC analyses of the diagnostic performance using different variables and their combinations for distinguishing true progression and pseudoprogression. The AUCs, sensitivity, and specificity of metabolites ratios, and rCBV were significantly larger than those of enhancement pattern of residual cavity wall (Figure 3, Table 3). With the cutoff rCBV value of 0.743, the AUC, sensitivity, and specificity of DSC-MRI for distinguishing true progression and pseudoprogression were 0.912, 76.5%, and 92.9%, respectively. The AUC, sensitivity, and specificity of the combination of Cho/Cr and Cho/NAA were 0.881, 88.2%, and 78.6%, respectively. The AUC and specificity of MRS + DSC were similar to those of CE + MRS + DSC combination model. However, the sensitivity and Youden index of MRS + DSC were relatively lower (Figure 3, Table 3). Interestingly, when we combined all MR variables, including enhancement patterns of residual cavity wall, rCBV, and metabolites ratios, the diagnostic performance was significantly improved (AUC 0.965, sensitivity 90.2%, and specificity 100.0%).

The agreement was excellent between the two neuroradiologists for evaluation of the functional MR variables, including rCBV (ICC 0.979, 95% CI: 0.966–0.987), ADC (ICC 0.995, 95% CI: 0.991–0.997), Cho/Cr (ICC 0.964, 95% CI: 0.941–9.978), and Cho/NAA (ICC 0.958, 95% CI: 0.932–0.974).

**Figure 4:**
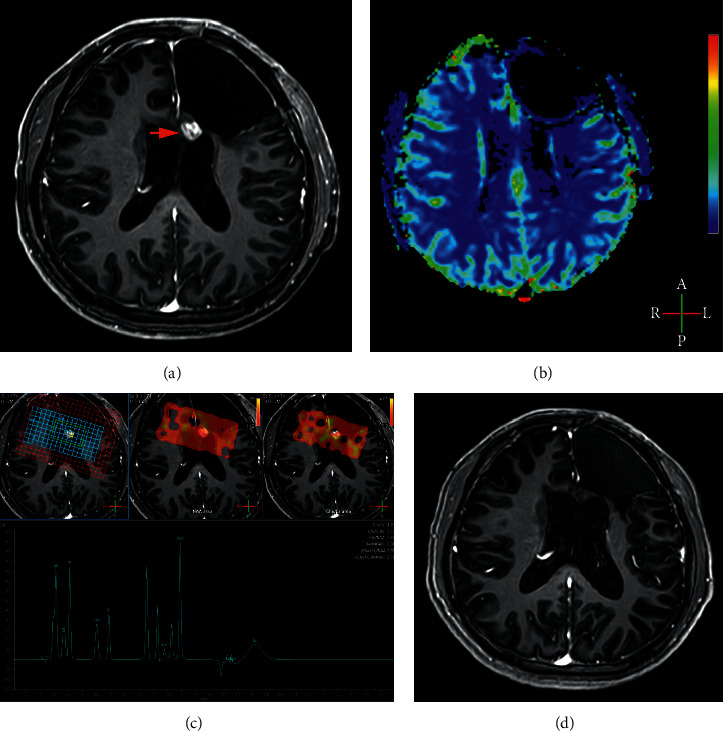
Example of pseudoprogression. A 21-year-old man with pathologically proven glioblastoma. A new enhancing lesion (arrow) developed in his left frontal lobe three months after completion of radiotherapy (a). No hyper-perfusion was detected on his DSC-MRI (b), with rCBV value of 0.71. MRS display Cho/Cr ratio of 1.20 and Cho/NAA 0.46 (c). The enhancing lesions in initial follow-up MRI disappeared in the MRI 23 months after completion of radiotherapy (d).

**Figure 5:**
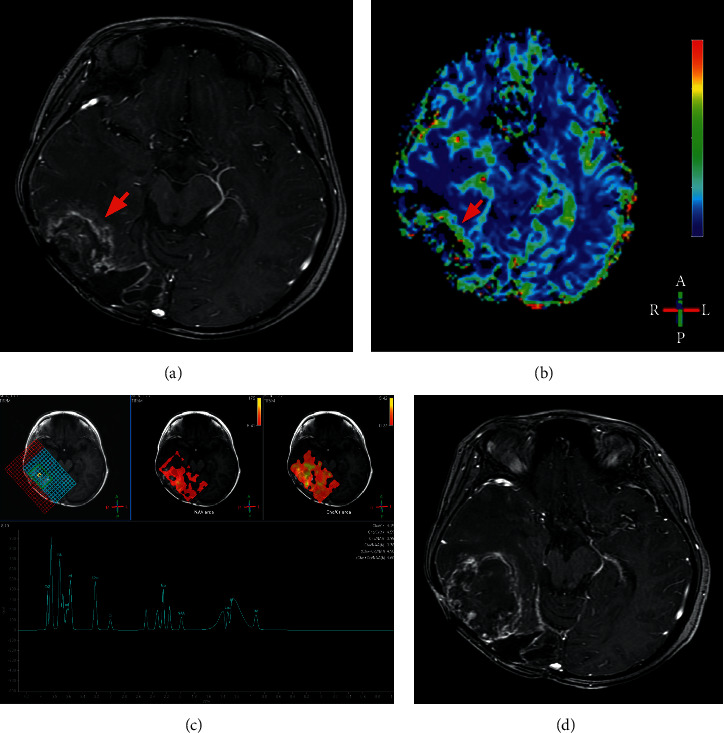
Example of true progression. An 18-year-old woman with pathologically proven glioblastoma. A new irregular ring enhancing lesion (arrow) developed in her right temporal-occipital lobe three months after completion of radiotherapy (a). Hyper-perfusion (arrow) was detected on her DSC-MRI (b), with rCBV value of 1.11. The MRS display Cho/Cr ratio of 4.15 and Cho/NAA 3.95 (c). The enhancing lesions enlarged significantly one month later (d). Tumor recurrence was confirmed pathologically after reoperation.

Figures 4 and 5 show the classic examples of true progression and pseudoprogression lesions.

## 4. Discussion

We applied DSC-MRI, along with conventional MRI sequences, DWI, and MRS, to assess the impact of perfusion parameter on the differential diagnosis of true progression versus pseudoprogression in patients with gliomas. Our results suggested that a combination of DSC-MRI, contrast enhanced T_1_W imaging, and MRS can greatly improve the diagnostic performance in distinguishing true progression from pseudoprogression.

Accurately and quickly distinguishing true progression from pseudoprogression in glioma patients after standard chemoradiotherapy remains a major clinical challenge [6]. Newly enhancement lesions or increase of the preexisting enhancement lesions, accompanied with mass-effect, as well as vasogenic cerebral edema, can be detected both in patients with true progression and in patients with pseudoprogression lesions [17]. Generally, longitudinal follow-up MRI over several months or stereotactic biopsy is needed for definitive diagnosis. In perfusion-weighted imaging, CBV could be used as an important biomarker for neoplastic vasculature [18, 19]. Our results support the hypothesis that DSC-MRI can add information for definitive diagnosis of glioma patients with gadolinium-enhancing lesions after treatment. In this study, rCBV is a valuable imaging marker for distinguishing true progression from pseudoprogression in glioma patients. Increased rCBV as surrogate biomarker of active growing tumor could potentially reduce the necessity for biopsy and their associated risks, thereby simplifying the posttreatment evaluation process and decreasing the care cost. On the other hand, DSC-MRI is the most common and available perfusion technique in the current commercial MR equipment. Thus, we suggest that DSC-MRI should be conventionally employed in the evaluation of posttreatment gliomas.

The DSC-MRI has been widely used in evaluation of the suspicious lesions in posttreatment glioma patients [5, 18–20]. The rCBV cutoff is important for identifying true progression. However, there is no consensus on the cutoff value of rCBV (0.71–5.01) for distinguishing true progression from pseudoprogression lesions [8, 18, 19, 21]. In a study of 44 glioblastoma patients, Blasel et al. found the cutoff value of 2.2 for rCBV yielded a sensitivity of 65% and a specificity of 71% [20], whereas, in the present study, with the cutoff value of 0.743 for rCBV, we achieved higher sensitivity (76.5%) and specificity (92.9%). This discrepancy may be attributed to the diversity of inclusion criteria and treatment regimens, as well as the lack of standardization in reference standard and imaging techniques [10, 21]. We enrolled gliomas patients with different grades (WHO grade II-IV) and with gross-total resection of tumors, and followed by standard therapy according to NCCN guideline. However, in Blasel et al.'s study [20], only glioblastoma patients (WHO grade IV) were recruited, including 4 patients who only had biopsy instead of tumor resection. Their therapy regimen was diverse, including Stupp's method, radiotherapy only, and second- and third-line therapy in some patients. Thus, the higher rCBV cutoff value in Blasel et al.'s study indicted higher mitotic activity and higher vascularity in their patients. In spite of the difference of cutoff value of rCBV, the diagnostic performance of our study was similar to other authors' [8, 19]. This indicted DSC-MRI, in spite of diverse rCBV cutoff value, can be used to improve the evaluation of progression in glioma patients after treatment [5].

Longitudinal change in rCBV may improve the discrimination between true progression and pseudoprogression lesions [10, 22]. The diagnostic performance, including AUC, sensitivity, and specificity, of rCBV difference between two sequential examinations was noticeably higher than that of initial rCBV in our study. Various pathological changes representing a wide range of local cerebral blood volume, including radiation-induced vasculopathy and necrosis, could coexist shortly after treatment. Thus, the rCBV value at the initial DSC-MRI may be less effective in discriminating the true progression from pseudoprogression lesions [10, 22]. However, the destiny of suspicious lesions becomes apparent over time. As the time goes on, increase in rCBV could be detected from those enhancing lesions that contain viable tumor cells, especially in the most active and aggressive region of the lesions.

MRS metabolites ratios provide useful information in distinguishing true progression from pseudoprogression lesions [8, 9, 23, 24]. Among several metabolic ratios [8, 24], Cho/Cr ratio was considered as the most favorable marker in differentiating between true progressions and pseudoprogression. Cho/Cr ratio achieved excellent sensitivity (91%) and specificity (95%) in a higher grade gliomas study [8]. However, in our study the sensitivities of MRS were relatively lower (51.0%) with the cutoff values of Cho/Cr ratio 2.475 and Cho/NAA ratio 2.115. A possible explanation for this discrepancy might be because we included lower-grade (WHO grade II and III) gliomas, which probably had lower metabolite concentration in the enhancing regions. Interestingly, our results showed that MRS index ratios achieved good diagnostic efficiency (AUC 0.881). Our findings support previous finding that MRS is a promising functional MR technique for the evaluation of treatment response in glioma [8]. However, we must keep in mind that using MRS ratios as a biomarker in the differential diagnosis has its intrinsic limitations; i.e., it is difficult to obtain universal cutoff values due to partial volume effect resulting from relatively large voxel size, and the ratios are vulnerable to the local field homogeneity, such as water, lipids, and surgical clips.

The role of DWI in differentiating true progression from pseudoprogression lesions remains ambiguous. Most studies showed the accuracy of ADC value in the differentiation was the lowest among all functional MR techniques [5, 8, 23]. The treatment effects, such as gliosis, coagulation necrosis, macrophages invasion, and demyelination, can reduce ADC value. Necrosis could be found in many recurrent tumor lesions [23]. Thus, the diagnostic value of DWI is weakened due to similar changes of diffusivity but quite different pathologic processes. In literatures, the sensitivity and specificity of ADC value in the differentiation varied widely (95% CI: 60–80%, and 95% CI: 77–93%, respectively) [8]. In our study, there was no significant difference in ADC between the two types of lesions. Similar results were presented in previous studies [23, 25]. However, in another previous study, the ADC value of recurrence lesions was significantly lower than that of non-recurrence lesions [5]. This discrepancy may be explained as bias from the relatively smaller sample size and inclusion of relatively lower-grade gliomas patients. Moreover, there was no consensus on the cutoff ADC value for this discrimination [5, 26, 27].

Since conventional morphologic imaging findings are often limited in differentiation of true progression from pseudoprogression lesions in gliomas, various advanced MR imaging modalities, including DWI, MRS, and DSC-MRI, can provide additional physiologic or metabolic information about posttreatment gliomas. However, the diagnostic accuracy of each technique is still limited and different [5, 8, 15, 28]. A multiparametric MR imaging has the potential to improve the overall diagnostic performance. In Matsusue et al.'s study [28], which enrolled 15 subjects, the diagnostic accuracy of combined multiparametric MRI (93.3%) was higher than those of ADC ratio (86.7%), rCBV (86.7%), and MRS (Cho/Cr and Cho/NAA) (84.6%). Our results confirmed the superiority of multiparametric MRI, because advanced imaging could provide more comprehensive information with respect to tumor pathophysiology. In the present study with relatively larger cohort with DSC-MRI, we further combined conventional morphologic imaging findings and advanced MRI data, especially the addition of DSC-MRI, which can be more reliable in differentiating true tumor progression from pseudoprogression. The combination of CE-T1WI, MRS, and DSC achieved the highest diagnostic performance (AUC, 0.965). Based on these results, we recommend that patients with suspicious enhancing lesions in conventional follow-up MRI should additionally receive advanced MR examinations, such as MRS and DSC-MRI.

Several limitations of this study should be mentioned. First, the number of the patients in this retrospective study was relatively small because we only recruited those patients with DSC-MRI and also with sufficient MRI follow-up data. Second, the final diagnosis of most patients (95.4%) in the current study was based on the follow-up data. Third, we measured CBV and DWI in a 2D mode, which could not reflect local pathologic changes completely. Fourth, although the longitudinal change in rCBV could improve the diagnostic performance, only 18 of 65 patients in our cohort had the sequential DSC-MRI data.

In conclusion, DSC-MRI, especially longitudinal change in rCBV, showed satisfactory diagnostic performance and can be a useful tool in distinguishing true glioma progression from pseudoprogression. Moreover, the combination of DSC-MRI and conventional morphological imaging features and other advanced MR techniques would further improve identifying the progression, which may greatly facilitate the individualized management of posttreatment glioma patients. This combination may even eliminate a long-term follow-up when a suspected gadolinium-enhancing lesion is found.

## Figures and Tables

**Table 1:** Clinical characteristics of 65 glioma patients.

Characteristics	Pseudoprogression (*n* = 14)	True progression (*n* = 51)	*t*/*X*^2^	*p* value
Mean age ± SD(years)	43.640 ± 14.372	47.290 ± 14.375	−0.842	0.403

Gender			0.022	0.881
Male	8	28		
Female	6	23		

1p19q codeletion(+) (*n* = 23)	1(33.33%)	4(21.05%)	—	0.814
Promoter of MGMT methylation (*n* = 12)	1(33.33%)	5(55.56%)	—	0.574
IDH mutation (*n* = 13)	1(50.00%)	3(27.27%)	—	0.178

WHO grading			—	0.451
II	1	3		
III	9	24		
IV	4	24		

KPS score			−1.427	0.154
Median	90.000	90.000		
95%CI	90.000–90.000	86.890–89.580		

T2-FLAIR mismatch			—	0.676
Yes	1	9		
No	13	42		

Enhancement of residual cavity wall			—	0.001
Thin-linear	11	12		
Thick-linear	1	10		
Nodular	2	29		

Total dose (GyRBE)			−0.503	0.615
Median	60	59.92		
95%CI	55.761–60.502	57.669–59.462		

SVZ involvement			—	0.204
Yes	7	36		
No	7	15		
ADC mean (mm^2^/s) (*n* = 54)	903.142 ± 491.652	523.000 (484.950–668.610)	−1.842	0.067

MGMT, O^6^-methyl-guanine methyl transferase; KPS, Karnofsky Performance Score; SVZ, subventricular zone; ADC mean, apparent diffusion coefficient mean; T2-FLAIR mismatch, the presence of a complete/near-complete hyperintense signal on T2-weighted (T2W) MRI sequences, in combination with a relative hypointense signal on fluid attenuation inversion recovery (FLAIR) MR sequences except for a hyperintense peripheral rim; Bold *p* value indicates statistically significant association, - indicates Fisher's exact test.

**Table 2:** Comparison of DSC and MRS variables.

	Pseudoprogression	True progression	*p* value
rCBV			**<0.001**
Median/mean	0.541 ± 0.154	1.094	
95%CI (range)	0.360–0.892	1.135–1.636	

Cho/Cr ratio			**0.001**
Median/mean	1.719 ± 0.664	2.520	
95%CI (range)	0.680–3.160	2.331–2.773	
Cho/NAA ratio	1.499 ± 0.495	2.414 ± 0.665	**<0.001**

rCBV, relative cerebral blood volume; Cho, choline; NAA, N-acetylaspartate; Cr, creatin; CI, confidence interval, Bold *p* value indicates statistically significant association.

**Table 3:** ROC curve analyses of diagnostic performance of various variables and their combinations.

Characteristics	AUC	95%CI	Sensitivity	Specificity	Youden index
CE	0.782	0.643–0.920	0.765	0.786	0.551
MRS	0.881	0.788–0.973	0.882	0.786	0.668
DSC	0.912	0.837–0.988	0.765	0.929	0.694
CE + MRS	0.930	0.867–0.993	0.882	0.857	0.739
CE + DSC	0.936	0.873–0.998	0.863	0.857	0.720
DSC + MRS	0.965	0.924–1.000	0.863	1.000	0.863
CE + MRS + DSC	0.965	0.923–1.000	0.902	1.000	0.902

CE, contrast enhancement pattern of residual cavity wall; MRS, Cho/Cr and Cho/NAA ratios; DSC, dynamic susceptibility contrast perfusion.

## Data Availability

The data used to support the findings of this study are available from the corresponding author upon request.
